# Association between the Munich Chronotype Questionnaire and Wrist Actigraphy

**DOI:** 10.1155/2018/5646848

**Published:** 2018-05-09

**Authors:** Jose Arturo Santisteban, Thomas G. Brown, Reut Gruber

**Affiliations:** ^1^Department of Psychiatry, McGill University, Montreal, QC, Canada; ^2^Addiction Research Program, Research Centre of the Douglas Mental Health, University Institute, Montreal, QC, Canada; ^3^Attention, Behavior and Sleep Lab, Douglas Mental Health University Institute, Montreal, QC, Canada

## Abstract

Chronotype refers to individuals' preferences for timing of sleep and wakefulness. It can be quantified by measuring the midpoint time between the start and end of sleep during free days. Measuring chronotype is helpful to diagnose circadian rhythm sleep-wake disorders. The Munich Chronotype Questionnaire (MCTQ) is a self-reported measure of chronotype that calculates the midpoint of sleep on free days based on self-reported bed and wake times. Self-reports of sleep are prone to bias. The objective was to examine the agreement between the MCTQ-derived midpoint and an objective measure obtained using wrist actigraphy. The sleep of 115 participants aged 18–34 (mean = 24, SD = 4.6) was monitored with actigraphy for 4 to 6 consecutive nights. The corrected midpoint of sleep on free days was derived from sleep start and end times on both free days and scheduled days. The corrected midpoint of sleep on free days as measured by the MCTQ was 4:56 (SD = 1 : 16) and by actigraphy was 4:51 (SD = 1 : 23). They were not significantly different (*t*_(87)_ = 0.66, *p* = 0.51). A strong correlation was found between these two measurements (*r*_(88)_ = 0.73, *p* < 0.001). The 95% limits of agreement were between −1:37:19 and 2:14:38. MCTQ and actigraphy provide similar results for the corrected midpoint of sleep on free days.

## 1. Introduction

Chronotype refers to preferences for timing of sleep and wakefulness [1, 2]. Chronotype reflects individual differences in circadian rhythms [3]. Determining chronotype is important as it affects many aspects of behavior and health, including sleep duration [4], cognitive performance [5], and psychopathology [6]. Determining chronotype is also helpful for the diagnosis and treatment of circadian rhythm sleep-wake disorders [7]. Objective measures can be used to estimate the relationship between the time point of internal biomarkers, such as dim light melatonin onset (DLMO) and core body temperature, and a zeitgeber (environmental cue, such as daylight) [8]. This relationship between the time points of the internal marker and external cue is called phase angle or phase of entrainment [9]. These biological measures exhibit high degrees of validity and reliability in assessing the circadian phase angle, but they are expensive and labor intensive and require high degree of subject participation [10]. Actigraphy may be used as an additional tool to estimate circadian phase angle [11]. Actigraphy is a noninvasive, objective measure of sleep that is reliable and valid compared to polysomnography in the measurement of sleep schedule and duration. However, it must be worn continuously at nights and requires participants' compliance.

Subjective measures of chronotype include questionnaires that ask about an individual's expressed preference for morning or evening activities. These questionnaires, such as the Morningness-Eveningness Questionnaire (MEQ) and Composite Scale of Morningness (CSM), can either classify individuals into categories (morning type, evening type, or neither type) based on such preferences or report chronotype on a continuum as a dimensional construct where an individual can have greater “morningness” or “eveningness” depending on where they fall on the spectrum. One such dimensional measure is the Munich Chronotype Questionnaire (MCTQ), a self-report questionnaire that collects information on an individual's habitual sleep schedule during work days and free days [12]. It estimates phase of entrainment by setting a reference point based on the reported sleep-wake cycle rhythm [8]. The reference point is the midpoint of sleep on free days and it is calculated by determining the time point between reported bedtime and wake-up time on nonwork days, where there is less influence from societal commitments (work, in this case) on sleep schedule. The MCTQ provides an inexpensive and easy way to calculate phase of entrainment, as evidenced by the strong correlations between the midpoint of sleep on free days with dim light melatonin onset (*r* = 0.68) [10], the results of the MEQ (*r* = −0.73) [12], moderate correlations with the results of the CSM (*r* = −0.58), and the midpoint of sleep on free days derived from sleep diaries (*r* = 0.44) [13]. One previous study found significant correlations between actigraphy-derived sleep start times and both corrected and uncorrected midpoint of sleep on free days (*r* = 0.20) and sleep end time and corrected midpoint of sleep on free days (*r* = 0.44) and midpoint of sleep on free days (*r* = 0.50) [14]. The paucity of data is a problem because self-reported sleep data is prone to bias [15], and it is not yet clear whether and to what extent the information provided by the MCTQ is accurate.

Current guidelines recommend that the circadian phase of individuals with circadian rhythm sleep-wake disorders be measured using an objective measure such as dim light melatonin onset or actigraphy [16]. Since it might not always be feasible for clinicians to objectively measure chronotype due to lack of access to actigraphy or DLMO timing, having a comparable measure that is easily accessible is essential.

The goal of this study was therefore to examine the association between the MCTQ, a self-reported questionnaire that measures chronotype based on sleep schedule, and objective measures of sleep schedule using actigraphy. Actigraphy determines wakefulness and sleep by measuring movement. It allows us to objectively measure sleep start and sleep end times which are the parameters used to determine phase of entrainment by the MCTQ. We hypothesized that the midpoint of sleep on free days as calculated by the MCTQ will be significantly and positively correlated with the midpoint of sleep on free days as calculated by actigraphy.

## 2. Methods

### 2.1. Participants

One hundred and fifteen participants were recruited from a larger study examining the role of sleep reduction and alcohol intake on driving performance. Inclusion criteria were (i) ages 18–24 and 30–34, (ii) having driven in the past 3 months and at least once per week; (iii) medication-free (except contraceptives for females); (iv) sleeps during regular night-time hours. Exclusion criteria are (i) Pittsburgh Sleep Quality Index > 5; (ii) performing shift-work; (iii) having health problem that contraindicates participation (i.e., attention-deficit hyperactivity disorder diagnosis, substance use disorder, and sleep problems and disorders); and (iv) being pregnant or breastfeeding.

### 2.2. Measures

*Munich Chronotype Questionnaire (MCTQ) [17]*. A self-report questionnaire collects information on an individual's habitual sleep schedule during scheduled (i.e., work) days and free days [12]. It was used to assess phase of entrainment by setting a reference point, “midpoint of sleep.”

MCTQ variables included the following: (a) sleep start, reported bedtime plus sleep onset latency; (b) sleep end, reported wake-up time; (c) sleep duration, total amount of time (in minutes) between sleep start and sleep end; (d) midpoint of sleep, the time point exactly in the middle between sleep start and sleep end times, and (e) corrected midpoint of sleep on free days, midpoint of sleep on free days plus half of the difference between the sleep duration on free days and a weighted average of sleep duration for both scheduled and free days (sleep duration of scheduled days times five and free days times two divided by seven). All variables, except for corrected midpoint of sleep on free days, were calculated for both scheduled days and free days.

*Actigraphy*. Actiwatch 2 (Philips Respironics) actigraphs were used to measure sleep. Actigraphy has been widely used to assess sleep and has been validated against polysomnography. In young adults at the threshold sensitivity used in this study, the agreement rate for epoch-by-epoch sleep-wake identification was reported to be 87.7% [18]. Actigraphy exhibits considerable test-retest reliability, with year-long intrasubject correlations of 0.73 for total sleep time, 0.93 for sleep onset latency, and 0.90 for sleep efficiency [19]. In the present study, each participant recorded bedtimes and wake times in a sleep log, and these times were used as the start and end times for the analyses. One-minute epochs were used to analyze actigraphic sleep data. For each 1-minute epoch, the total sum of activity counts was computed. If they exceeded a threshold (threshold sensitivity value = mean score in active period/45), then the epoch was considered waking. If it fell below that threshold, then it was considered sleep.

Scoring was based on the Society for Behavioral Sleep Medicine guide to actigraphy monitoring [20]. As recommended by the guide, nights were not scored if reported sleep start times and end times were discordant from actigraphic measurements for more than one hour.

The actigraphic data were analyzed using sleep software (Actiware Sleep 6.1, Philips Respironics). The analyzed parameters included the (a) sleep start, which was the beginning of sleep; (b) sleep end, the end of sleep; (c) sleep duration, the total amount of time (in minutes) between sleep start and sleep end; (d) midpoint of sleep, the time point equidistant between sleep start time and sleep end time, and (e) corrected midpoint of sleep on free days, midpoint of sleep on free days minus half of the difference between the sleep duration on free days and average sleep duration for all days.

To calculate the averages of either scheduled (i.e., work or school) or free days, participants self-reported on the sleep log if the day was scheduled or free. Averages were only calculated if there were at least two nights for either scheduled days or free days.

### 2.3. Procedure

Data collection was conducted in two sessions. On the first session, a questionnaire was administered to collect demographic data and the MCTQ was administered to measure chronotype. Participants were then given an actigraph and instructed to wear it on the nondominant hand at bedtime for six consecutive nights, including both week and weekend nights, as well as during free and scheduled (i.e., work) nights. Six nights later, on the second session, the actigraph was collected and the sleep data was analyzed. The study was approved by the by the Research Ethics Board of Douglas Mental Health University Institute (Montreal, Canada). Written informed consent was provided prior to data collected.

### 2.4. Analyses

Means and standard deviations were calculated for actigraphic sleep measures and the MCTQ. Normality of distribution for each of the variables was tested with the Shapiro-Wilks test. Paired two-tailed *t*-tests were used to compare means between MCTQ measures and actigraphic sleep measures. Pearson's correlations were used to measure associations between MCTQ and actigraphy-derived measures of sleep start, sleep end, sleep duration, and midpoint of sleep. Differences were analyzed with the Bland-Altman method [21]. For each participant, the differences between the MCTQ- and the actigraphy-derived corrected midpoint of sleep on free days were calculated, as well as the mean of these measures. Bland-Altman plots were used to depict the difference between the means of the two measures and the limits of agreement were calculated as 1.96 standard deviations of the difference from the mean. A linear regression comparing the difference between the measures (MCTQ-derived corrected midpoint of sleep on free days minus actigraphy-derived corrected midpoint of sleep on free days) as the dependent variable and the mean of the two measures as the independent variable was used to identify biases in the difference for participants with earlier or later types. Results were considered statistically significant when *p* < 0.05. SPSS 22 for Windows (IBM) was used for all statistical analyses.

#### 2.4.1. Power Analyses

Power analysis to detect difference between two dependent means was conducted using the software package, GPower [22]. A priori analyses with an alpha significance level of 0.05 and a power of 0.80 determined that 403, 67, and 28 participants are needed to detect small, medium, and large effect sizes, respectively. Sensitivity analyses, with an alpha significance level of 0.05 and a power of 0.80, determined that minimum detectable effect size (*d*) was 0.51 for the midpoint of sleep on free days sample (*n* = 101) and 0.51 and for the corrected midpoint of sleep on free days sample (*n* = 88).

## 3. Results

Table 1 shows the demographic characteristics of this sample.

The range of nights utilized was four to six nights for participants to be included in analyses (115 participants were included in the analyzes, of which 90 participants had six nights of data, 23 had five nights of sleep data, and 2 had four nights of sleep data). 101 participants had at least two nights of sleep data on free days necessary to analyze free days and 101 participants had at least two nights of sleep data on scheduled days necessary to analyze scheduled days, while 88 had both at least two nights of sleep data for free days and two nights of sleep for scheduled days required to analyze the corrected midpoint of sleep. 32% of the sample (37 participants) reported not using an alarm clock on free days. The data for all variables was normally distributed.

MCTQ-derived midpoint of sleep on free days was 5:00 (SD = 1 : 15), while that measured by actigraphy was 4:55 (SD = 1 : 19). There was no significant difference between the means of these two groups (*t*_(100)_ = −0.95, *p* > 0.05, *d* = 0.06). A strong correlation was found between these two measurements (*r*_(101)_ = 0.71, *p* < 0.001).

The corrected midpoint of sleep on free days as measured by the MCTQ was 4:56 (SD = 1 : 16) and as measured by actigraphy was 4:51 (SD = 1 : 23). No significant difference was found between the means of these two groups (*t*_(87)_ = 0.66, *p* = 0.51, and *d* = 0.06). A strong correlation was found between these two measurements (*r*_(88)_ = 0.73, *p* < 0.001). 

When correlations were analyzed by sex or age group, the correlation remained statistically significant for each of the groups.

**Figure 1:**
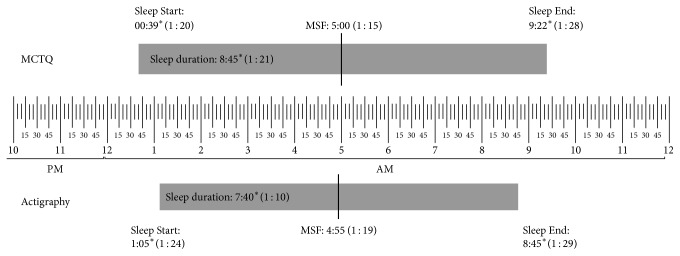
Means and standard deviations of MCTQ and actigraphy on free days. MCTQ: Munich chronotype questionnaire. MSF: midpoint of sleep on free days. SD: standard deviation. Significant differences between the two measures were marked with an asterisk.

**Figure 2:**
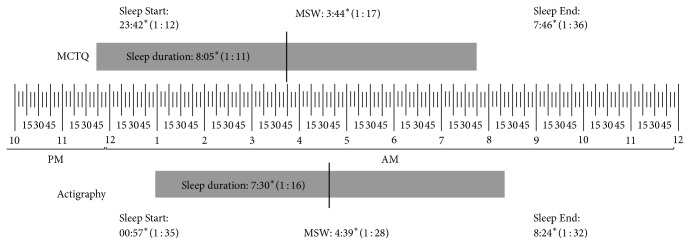
Means and standard deviations of MCTQ and actigraphy on work days. MCTQ: Munich chronotype questionnaire. MSW: midpoint of sleep on work days. SD: standard deviation. Significant differences between the two measures were marked with an asterisk.

Detailed results for the sleep schedule and sleep duration, as obtained from the MCTQ and actigraphy, are presented in Figure 1 for free days and in Figure 2 for work days.  Table 2 presents the correlations obtained between the MCTQ and actigraphy for the sleep schedule and duration on both work and free days.

**Figure 3:**
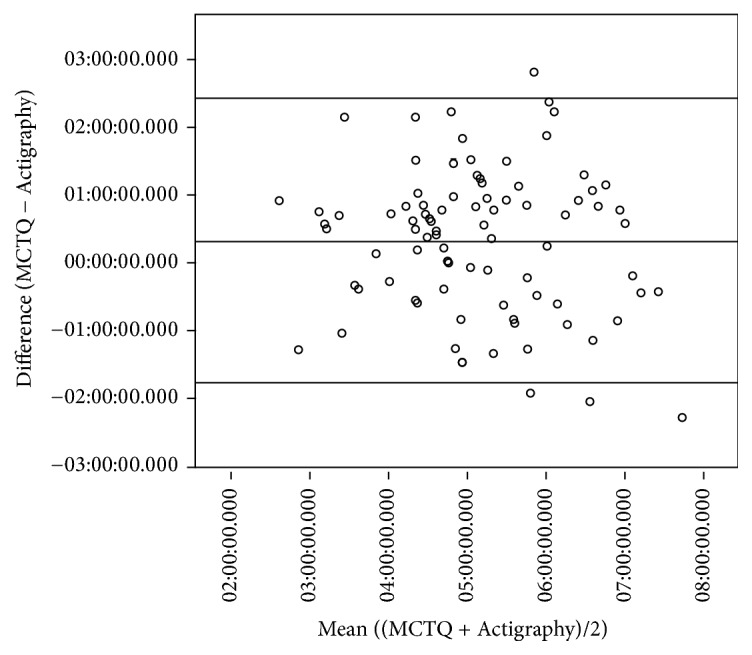
Bland-Altman plot comparing the MCTQ and actigraphy-derived corrected midpoints of sleep. Upper and lower lines present the 95% limits of agreement. Middle line represents the mean difference between the measures.

Figure 3 presents a Bland-Altman plot for the corrected midpoint of sleep on free days. The 95% limits of agreement between the two measures were between −1 : 37 : 19 and 2 : 14 : 38. The mean of the two measures was not a significant predictor of the difference between the two measures (*F*_(1,87)_ = 1.28, *p* = 0.26). Hence, the difference between the measures is not significantly greater or lesser for people with later or earlier midpoints of sleep.

## 4. Discussion

The objective of this study was to compare the MCTQ-derived midpoint of sleep on free days with those calculated using actigraphy. The MCTQ- and actigraphy-derived midpoints of sleep were significantly and strongly positively correlated, and the mean MCTQ- and actigraphy-derived midpoints were not significantly different. The corrected midpoint of sleep on free days as measured by the MCTQ and by actigraphy was also significantly and strongly correlated, and the means were also not significantly different. Differences between the two measures were not larger (or smaller) for people with later (or earlier) midpoints of sleep. Collectively, the present data provide evidence of strong agreement on average between MCTQ-derived measures of corrected midpoint of sleep on free days and those derived from actigraphy. Thus, these findings provide further support for the use of the MCTQ to assess chronotype for both research and epidemiological purposes.

However, there is one caveat when comparing the MCTQ- and actigraphy-derived corrected midpoints of sleep on free days. The limits of agreement (where 95% of differences between these two measures are from the mean of both of these measures) range from 1 hour and 37 minutes earlier and 2 hours and 15 minutes later (3 hour and 52 minute range). The range for 95% of subjects is wide enough that the differences would be clinically significant. Similarly, a previous study comparing the MCTQ-derived midpoint of sleep in free days and DLMO found that the differences between them could be in a 4-hour range [10]. Due to the wide range in the limits of agreement of the assessments of the two measures, the MCTQ cannot be used to estimate an objectively measured corrected of midpoint of sleep in an individual. These differences may be due to the MCTQ reporting ideal sleeping patterns, rather than actual sleeping patterns. Therefore, the self-reported responses to the MCTQ do not necessarily accurately reflect actual, objectively measured sleep behavior in one subject. These results support the conclusion by Kantermann et al. [10] that the MCTQ-derived corrected midpoint of sleep on free days should not solely be used to time treatment of circadian rhythm sleep-wake disorders.

While significant correlations between MCTQ- and actigraphy-derived measures for sleep start, sleep end, and sleep duration were found, there were also significant differences between the means. Therefore, for these other measures, the MCTQ, as with other subjective measures of sleep behavior, is prone to bias when compared to an objective measure such as actigraphy [15]. Sleep times were more accurately reported on free days than on work days. Participants may have responded the MCTQ with their idealized sleep schedule for work days, rather than accurately describing their actual sleep schedule.

Previous studies that compared the MCTQ-derived midpoint of sleep with dim light melatonin onset found that they were associated [10]. Thus, it may be interesting to compare how both the MCTQ and actigraphy compare to dim light melatonin onset. It may also be of practical use to compare the sensitivity and specificity of the MCTQ and actigraphy for diagnosing circadian rhythm sleep-wake disorders in a clinical population.

A strength of this study, compared to previous ones using comparing the MCTQ with dim light melatonin onset [10, 23], is the larger sample size. In terms of limitations, the present study may suffer from reduced generalizability due to the characteristics of the sample. Participants were excluded if they had poor sleep based on the Pittsburgh Sleep Quality Index. Therefore, these results may not generalize to a population with poor sleep quality. Participants were between 18 and 34 years of age, and the majority (77.3%) were students, although the results remained significant when occupation was included as a covariate. It is possible that a study with older or employed participants could yield different results, as circadian preference changes with age and scheduling from employment changes sleep behavior. A limitation of this study is that wrist actigraphy was only used for 4–6 nights, whereas the MCTQ covers 7 nights. While most participants reported using an alarm clock on free days, removing these participants did not change the results. Another limitation of the study is related to the fact that the definition of free day in this study would include individuals who awaken not due to work schedule but due to external factors such as children or pets [24].

## 5. Conclusion

MCTQ- and actigraphy-derived corrected midpoints of sleep on free days are on average the same between the two measures. These findings support the use of the MCTQ to assess chronotype, but not to time treatment that requires a precise assessment of chronotype. The MCTQ self-reported sleep schedules do not necessarily represent actual behavior.

## Figures and Tables

**Table 1:** Demographic characteristics of sample.

	Mean (SD)
*Age (years)*	24 (4.6)

*Education level (years)*	16 (2.19)

	*N* (%)

*Sex*	

Male	51 (44%)
Female	64 (56%)

*Race*	

White	86 (74.0%)
Chinese	3 (2.6%)
Black	10 (8.5%)
Latin American	3 (2.6%)
Arab	2 (1.7%)
West Asian	1 (0.9%)
Others	10 (9.4%)

*Income*	

None	11 (9.4%)
$1–5,999	26 (23.1%)
$6,000–19,999	54 (46.9%)
$20,000–39,999	17 (14.5%)
	7 (6.0%)

*Occupation*	

Work and study	65 (56.5%)
Study	24 (20.8%)
Work	16 (13.9%)
Unemployed	4 (3.5%)
Unstable	6 (5.2%)

*Marital status*	

Single	88 (76.1%)
Married	24 (21.4%)
Divorced	3 (2.6%)

**Table 2:** Correlation between actigraphic sleep data and self-reported data on the Munich Chronotype Questionnaire (MCTQ).

Measure	Correlation	
	Work day	Free day
Sleep start time	*r*_(101)_ = 0.34^*∗∗*^	*r*_(101)_ = 0.50^*∗∗∗*^
Sleep end time	*r*_(101)_ = 0.50^*∗∗∗*^	*r*_(101)_ = 0.67^*∗∗∗*^
Sleep duration	*r*_(101)_ = 0.17	*r*_(101)_ = 0.38^*∗∗∗*^
Midpoint of sleep	*r*_(101)_ = 0.64^*∗∗∗*^	*r*_(101)_ = 0.71^*∗∗∗*^

^*∗*^ *p* < 0.05; ^*∗∗*^*p* < 0.001; ^*∗∗∗*^*p* < 0.000. Correlations remained significant when controlling for gender, age, income, education level, race, marital status, and occupation.

## Data Availability

The data used to support this study is part of an ongoing project. Access to these data will be considered by the author upon request.
